# Journey mapping paediatric oncology care in Saudi Arabia: A mixed-methods study

**DOI:** 10.4102/hsag.v30i0.3110

**Published:** 2025-11-30

**Authors:** Febe V. Lacson, Dorien Wentzel

**Affiliations:** 1 Department of Nursing, College of Health Sciences, University of KwaZulu-Natal, Durban, South Africa; 2 Department of Pediatric Oncology, King Faisal Specialist Hospital & Research Centre, Madinah, Saudi Arabia

**Keywords:** journey mapping, paediatric oncology, mixed methods, family experiences, Saudi Arabia

## Abstract

**Background:**

Because of the global increase in childhood cancer, patient journey mapping was utilised to improve the treatment experiences and outcomes for paediatric oncology patients and their families.

**Aim:**

The aim of this study is to describe the process of developing a patient journey map for paediatric oncology care and to present insights from families’ experiences across different stages of the care pathway.

**Setting:**

The study was conducted at a tertiary care hospital in the Kingdom of Saudi Arabia, which provides oncology services for paediatric patients with malignant disorders.

**Methods:**

An explanatory sequential mixed-methods design was utilised. Quantitative data were collected from 134 family members using the Press Ganey Inpatient Survey. Data were analysed using SPSS 103 version 27. Nine family members were interviewed, data were analysed using manifest content analysis. The results were integrated to develop a descriptive care map. To refine the journey map, a modified Delphi method was applied. The initial design was shared with a Delphi panel comprising senior paediatric oncology staff and consensus was achieved.

**Results:**

A survey of 134 questionnaires revealed a 77% response rate. Qualitative insights from the nine interviews revealed six key themes: care from nurses, care from physicians, positive experience, negative experience, improvement in care and feelings summarising the journey.

**Conclusion:**

The study provided valuable information of patients’ families’ satisfaction with the experiences throughout the oncology units and simultaneously highlighted areas for improvement.

**Contribution:**

This study visualises a patient’s care journey through a complex system, allowing healthcare providers to fill gaps in care processes.

## Introduction

Cancer incidence continues to rise globally, with approximately 19.3 million new cases reported in 2020 (Sung et al., 2021; You & Henneberg 2017). Childhood cancer accounts for a significant portion of these cases, with an estimated 400 000 children and adolescents diagnosed annually (World Health Organization [WHO] 2021). In Saudi Arabia, where children under 15 years comprise 30.3% of the population, paediatric oncology remains a critical area of healthcare (Saudi Commission for Health Specialists 2021). Historical data indicated that paediatric oncology cases constituted approximately 8% of cancer diagnoses between 1999 and 2008 (Al-Mutlaq et al., 2015). More recent findings estimate an annual age-specific cancer incidence rate of 99.83 per million in Saudi children (Belgaumi et al., 2019). The rising burden of paediatric cancer necessitates systematic approaches to optimise healthcare delivery, particularly as childhood and adolescent malignancies are poised to surpass infectious diseases as the leading cause of disease-related mortality (WHO 2021).

Understanding the treatment experiences of paediatric oncology patients and their families is fundamental to improving healthcare outcomes. Patient journey mapping (PJM) is an effective strategy to enhance patient-centred care by visualising the entire care trajectory and identifying pain points (Arias et al., 2020). Patient journey mapping is instrumental in capturing the emotional, cognitive and social dimensions of healthcare interactions (Hall et al., 2015). This technique enables healthcare providers to re-evaluate their approach to treatment from the patient’s perspective, thereby enhancing communication, reducing uncertainties and fostering an improved care experience (Thamrin 2020).

The concept of journey mapping encompasses both the functional and emotional aspects of care delivery, detailing patient behaviours, motivations and attitudes across various touchpoints (Trebble et al., 2010). These touchpoints represent critical interactions between patients, families and healthcare providers, influencing clinical outcomes (Arias et al., 2020). Research suggests that healthcare professionals, because of their specialised roles, often engage with patients in a fragmented manner, focusing only on specific treatment phases. Consequently, a comprehensive map that outlines the patient’s entire care journey is necessary to improve coordination and holistic care provision (Bolz-Johnson, Meek & Hoogerbrugge 2020; Joseph, Kushniruk & Borycki 2020).

Paediatric oncology patients and their families frequently experience distress and uncertainty, particularly when information regarding treatment and prognosis is incomplete or inconsistently communicated (Keiza, Chege & Omuga 2017). The impact of childhood cancer extends beyond the patient, affecting parental mental health, family dynamics and siblings’ well-being (Jang et al., 2023; Vrontaras 2018). Parents must balance caregiving responsibilities with concerns about relapse, treatment side effects and the long-term implications of cancer (Erker et al., 2018; Nasab & Foroghi 2016). Journey mapping can mitigate these challenges by delineating expected transitions in care and available resources, thereby fostering preparedness and resilience among affected families (Pedersen et al., 2020; Thienprayoon et al., 2022).

Despite its benefits, PJM remains relatively underutilised in paediatric oncology, particularly in Middle Eastern healthcare systems (Munday et al., 2019). Existing studies have demonstrated that PJM enhances care continuity, reduces inefficiencies and improves communication between patients and providers (Devi et al., 2020; Saragosa et al., 2023). Research in Saudi Arabia has primarily focused on journey mapping for chronic conditions such as hypertension and dyslipidaemia (Amir et al., 2023) or chronic pain (Bahlas et al., 2021), highlighting substantial gaps in critical patient experiences. Given Saudi Arabia’s commitment to improving oncology care, integrating PJM into paediatric oncology services presents a strategic opportunity to refine care models and enhance patient–family engagement.

The article aims to report on the methodological process and outcomes of developing a patient journey map for paediatric oncology care and present insights from families’ experiences across different stages of the care pathway. The findings will contribute to a structured framework for delivering patient-centred paediatric oncology care in Saudi Arabia, ensuring a more cohesive and empathetic treatment experience.

## Research methods and design

### Study design

This study employed an explanatory sequential mixed-methods design, integrating both quantitative and qualitative data collection. The approach was selected to provide a comprehensive understanding of the research problem by initially collecting and analysing quantitative data, followed by qualitative data to explain and expand on the findings (Creswell 2014; Schoonenboom & Johnson 2017). The mixed-methods design is underpinned by pragmatism, which emphasises problem-solving through the interplay of knowledge and action (Allemang, Humphries & Barber 2022; Okesina 2020).

The quantitative phase involved a structured survey using the Press Ganey Inpatient Survey (PGIS) to measure family satisfaction with paediatric oncology care. The qualitative phase included semi-structured interviews with family members to gain deeper insights into their experiences. The integration of data followed a three-stage process:

1. *Connecting* – The results of the quantitative phase informed the participant selection for qualitative interviews.
2. *Building* – The qualitative phase was designed to explain the quantitative results.
3. *Merging* – Both datasets were analysed to identify complementary and divergent findings (Fetters, Curry & Creswell 2013).

### Study setting

The study was conducted at a newly commissioned central and tertiary care hospital in Madinah, Saudi Arabia, which serves a population of approximately 1.5 million people. The hospital specialises in oncology care and provides advanced treatment modalities, including chemotherapy, immunotherapy and targeted therapy.

The facility includes an 18-bed infusion area for outpatient chemotherapy, with six beds dedicated to paediatric oncology patients, a 16-bed inpatient paediatric oncology and haematology unit, admitting children diagnosed with leukaemia, lymphomas and solid tumours, and a dedicated paediatric oncology outpatient clinic for follow-up care post-discharge.

### Data collection process

Data collection was conducted in three phases, integrating quantitative and qualitative methods to ensure a comprehensive understanding of family experiences in paediatric oncology care (Table 1).

**TABLE 1:** Overview of the research process.

Phase	Objective	Participants	Methods or tools
Phase 1: Quantitative	Identify key stages and touchpoints; measure family satisfaction	Senior oncology staff (workshop), family members (survey)	Workshop discussion; Press Ganey Inpatient Survey
Phase 2: Qualitative	Explore families’ experiences at each stage of care	Family members	Semi-structured interviews
Phase 3: Integration and refinement	Develop and validate the patient journey map	Researcher + senior oncology staff	Mixed-method integration; modified Delphi process

Participants provided informed consent before data collection, with confidentiality and anonymity ensured throughout the study.

#### Phase 1: Workshop – Identifying key journey stages

The study commenced with a researcher-led workshop conducted in the inpatient oncology unit’s boardroom, with six purposively selected senior oncology staff members (four paediatric oncology nurses and two physicians; ≥ 8 years’ experience). Junior staff and trainees were excluded. The objective was to outline and define the key stages of a paediatric oncology patient’s journey and identify touchpoints where patients and families interacted with health services.

The workshop followed a structured discussion format, allowing participants to map each stage, discuss existing care processes and suggest areas for improvement. Discussions were audio-recorded with participant consent, and data from the workshop were transcribed for thematic analysis. The insights gained were subsequently used to refine the next phases of data collection, ensuring alignment with real-world care delivery practices (Table 2).

**TABLE 2:** Workshop layout for mapping the paediatric oncology patient journey.

Workshop aim	To determine the typical journey of a paediatric oncology patient from pre-diagnosis to aftercare through the oncology units.
Focus areas	Description
Naming the persona	Define a representative ‘patient persona’ for the care map
Identifying stages	Outline the key stages or broad activities in the patient’s journey
Touchpoints	Describe interactions at each stage between patients or families and healthcare providers
Timeline	Indicate duration of each touchpoint, time gaps between touchpoints and overall journey length
External influences	Identify non-health system factors affecting the journey (e.g. patient’s distance from facility)
Internal influences	Identify health system-related factors impacting the journey (e.g. ward renovations)
Journey illustratioin	Produce a visual map showing stages, timeline and touchpoints

#### Phase 2: Quantitative data collection – Press Ganey Inpatient Survey

The PGIS, a validated and widely used instrument in patient experience research, was employed to collect quantitative data on family satisfaction with paediatric oncology care. This open-access tool has demonstrated excellent internal reliability, with a reported Cronbach’s alpha of 0.98 (Press Ganey Associates Inc. 2010). In this study, a pilot test conducted with 10 participants yielded a Cronbach’s alpha of 0.89, confirming the questionnaire’s high reliability in the study context.

**Participant recruitment and survey administration:** Family members of recently discharged paediatric oncology patients were recruited for participation. Eligible participants were approached by the ward nurse and provided with an information sheet in English and Arabic, explaining the purpose of the study. They were informed about their right to withdraw at any time, and assurances were given regarding the confidentiality of their responses.

For non-English-speaking participants, an Arabic interpreter was available to facilitate communication. Participants self-administered the surveys, which were then returned in sealed envelopes and deposited in a secured collection box at the nurse station, ensuring anonymity.

A total of 130 questionnaires were distributed, with 100 completed and returned. The collected data were analysed using SPSS Version 27, focusing on descriptive statistics and examining associations between socio-demographic variables and satisfaction scores.

#### Phase 3: Qualitative data collection – Semi-structured interviews

The qualitative phase was designed to gain a deeper understanding of family experiences by exploring their perspectives in greater detail.

**Participant selection:** Participants for semi-structured interviews were selected from those who had completed the PGIS survey and had indicated willingness to participate in follow-up research. A purposive sampling approach was employed to ensure a diverse representation in terms of demographics, the length of hospital stay and the child’s diagnosis.

**Interview process:** Interviews were conducted in a private consultation room within the hospital to ensure confidentiality and participant comfort. Each interview lasted approximately 30 min–45 min. Before the session, participants provided written informed consent for audio recording.

Interviews were conducted in either English or Arabic, depending on the participant’s preference, and an Arabic interpreter was available when needed. A semi-structured interview guide was used to facilitate discussions, covering several key areas. Participants were asked to describe their experiences at different stages of care, including diagnosis, treatment, discharge and follow-up. They were also invited to discuss challenges encountered, factors that facilitated their journey and suggestions for improving paediatric oncology services.

A total of nine family members participated in the interviews as data saturation and redundancy were achieved.

**Integration of data and triangulation:** The final stage of data collection involved integrating quantitative survey results and qualitative interview findings through the modified Delphi approach (Ly, Runacres & Poon 2021). A panel of six senior oncology staff members reviewed the emerging themes and the proposed journey map framework. Their feedback was incorporated, leading to the refinement of the final care map. Consensus was ultimately achieved, ensuring the rigour and validity of the study’s findings.

### Data analysis

This study employed an explanatory sequential mixed-methods design, integrating both quantitative and qualitative approaches to provide a comprehensive analysis. The quantitative data were collected using the PGIS and analysed with IBM SPSS Version 27.0. Descriptive statistics, including frequencies, percentages, means and standard deviations, were used to summarise key variables. To examine associations between socio-demographic factors and satisfaction scores across different stages of the paediatric oncology journey, Pearson’s chi-square test was conducted, with statistical significance set at *p* < 0.05.

For the qualitative phase, semi-structured interviews were conducted, transcribed verbatim and translated into English when necessary. The data were analysed using manifest content analysis, guided by Creswell’s (2014) framework. The analysis followed a structured process, beginning with familiarisation, where transcripts were reviewed multiple times to ensure accuracy and completeness. This was followed by the coding phase, where descriptive labels were assigned to significant statements. The identified codes were then categorised into broader themes and subthemes. Finally, an interpretative phase was conducted to explore emerging insights, contradictions and unique perspectives within the data.

To enhance the depth and validity of the study, a comparative approach was used to integrate the quantitative and qualitative findings. The connection between the two datasets was established by using the quantitative results to guide the selection of qualitative interview participants. Additionally, the qualitative insights were utilised to explain unexpected findings from the quantitative analysis, ensuring a more nuanced understanding of the data. Finally, both datasets were merged to identify converging and diverging themes, strengthening the study’s conclusions and providing a more holistic depiction of the paediatric oncology care experience. This multi-step analysis ensured methodological rigour and enhanced the reliability and validity of the study’s findings.

### Rigour in mixed-methods study

Rigour in this mixed-methods study was ensured through methodological and analytical strategies applied to both the quantitative and qualitative phases. For the quantitative phase, the PGIS, a widely validated instrument, was used to collect data. The tool demonstrated excellent internal consistency, with a reported Cronbach’s alpha of 0.98. A pilot study conducted with 10 participants further supported its reliability, yielding a Cronbach’s alpha of 0.89. In the qualitative phase, rigour was maintained by employing strategies to ensure credibility, dependability, transferability and confirmability. Credibility was enhanced through data saturation, member checking and verbatim transcription. Dependability was established via ethical approvals from two independent committees and consistency in data collection. Transferability was achieved by providing rich, contextual descriptions of the setting, participants and study design. Confirmability was upheld by maintaining objectivity and supporting findings with direct participant quotations. The integration of both datasets through triangulation further strengthened the trustworthiness and validity of the study’s conclusions.

### Ethical considerations

The study adhered to strict ethical protocols to protect participants’ rights and ensure compliance with research ethics. Ethical approval was obtained from the UKZN Biomedical Research Ethics Committee (BREC), approval number BREC/000046791/2022, and from the Hospital and Research Centre Institutional Review Board (IRB) in the Kingdom of Saudi Arabia, approval number RAC/M2022-0004. Gatekeeper permission was also secured from the hospital management before participant recruitment.

Participation in the study was entirely voluntary, with informed consent obtained from all participants. To ensure accessibility, all study documents, including consent forms, were provided in both English and Arabic. Participants were informed of their right to withdraw from the study at any stage without consequences. Anonymity was maintained by assigning unique numerical identifiers to survey responses and interview transcripts.

Recognising the potential emotional impact of discussing paediatric oncology experiences, the study implemented protective measures for vulnerable participants. A hospital psychologist was made available for referrals in case of distress, and participants had the option to pause or withdraw from interviews.

To ensure data security, all research data were stored in password-protected digital files and securely archived hard copies. In accordance with institutional guidelines, research data will be retained for 5 years before being permanently deleted or destroyed.

## Results

As the primary aim of this article is to report the development of the patient journey map, the results begin with a detailed description of this process.

### Development of the patient journey map

#### Stage 1: Initial framework

The multi-stakeholder workshop, which included four senior paediatric oncology nurses and two paediatric oncology physicians, was conducted to outline the key stages of a paediatric oncology patient’s journey. The discussion highlighted areas needing better care coordination, including delays in diagnosis and post-discharge follow-up. The outcome of the workshop is represented in Table 3 with the transcription of journey mapping.

**TABLE 3:** Transcription of the patient journey mapping.

Stage	Touchpoints	Tasks or activities	Timeline	Internal influences	External influences
Pre-diagnosis	Symptom identification, referral and initial consultation with the oncologist	Parents or child notice symptoms; family visits clinic and gets referred to oncologist for further evaluation; initial consultation with oncologist	3–4 weeks from symptoms	Arrangement of work up; lack of slots; lack of manpower; lack of specified equipment or facility	Acceptance and eligibility criteria; living far; lack of knowledge; reluctance to go; spiritual reasons
Diagnosis	Diagnostic testing and disclosure	Conduct diagnostic tests (blood tests, imaging, scans, biopsy); oncologist discloses diagnosis; plan for hospitalisation; family receives call for admission	1 week	Delayed test results; send out labs; equipment malfunction; bed availability	Denial by parents; family domestic issues
Treatment	Treatment planning, treatment administration and monitoring progress	Oncology team designs treatment plan (chemo, radiation, surgery, immunotherapy, etc.); family counselling; central line access; informed consents; begin treatment; radiological procedures and daily labs; adjustments to treatment based on response, side effects, well-being; emotional/psychosocial support, nutritional support; visitors and family support; MDT rounding	Dependent on protocol and Dx (leukaemia: 3 years; solid tumour: 6 months; inpatient admission for leukaemia: 3 weeks–1 month)	Unavailability of antineoplastic drug; lack of manpower; bed availability	Global shortage of specific chemo drug
Discharge	Completion of treatment and discharge	Discharge planning (home care, medications, diet, appointments); physician prepares discharge summary and current medical status; care plan given to caregiver	24 h but planning starts on admission	Delay in discharge decision; patient’s medical condition; social issues; family refusal; unavailability of housing	Home is far from hospital facility
Post-discharge follow-up	Follow-up appointments, physical rehabilitation, psychological support, long-term monitoring and survivorship care plan	Regular scheduled visits with oncology team; central line care at home; outpatient treatment continuation; regular screenings and check-ups; lifestyle and survivorship plan provided (late effects, emotional health, growth and development)	May vary (leukaemia: 3 years)	Lack of palliative care team; unavailability of transplant services	Lack of community support; lack of interest in follow-up; social issues

### Integration of quantitative and qualitative data

The journey map was constructed by integrating survey responses from caregivers (quantitative phase) and in-depth interviews (qualitative phase). This process ensured that both statistical trends and personal experiences were captured.

#### Quantitative phase: Statistical foundation of the journey map

The PGIS was completed by 134 caregivers, yielding a **77%** *response rate*. Key findings included:

- *High satisfaction* with nursing care (90%) and physician communication (92%).
- *Moderate satisfaction* with emotional support services (76%), indicating a need for better psychological support.
- *Lowest ratings* were recorded in discharge education (68%) and meal quality (4.19 mean score), highlighting areas requiring improvement.

Table 4 presents a breakdown of satisfaction scores across different care domains.

**TABLE 4:** Participants’ satisfaction with the domains of care (*N* = 100).

Items	Mean	s.d.	Very poor		Poor		Fair		Good		Very good	
			*n*	%	*n*	%	*N*	%	*n*	%	*n*	%
**Domain 1: Admission**												
Speed of admission process	4.66	0.517	0	0.0	0	0.0	2	2.0	30	30.0	68	68.0
Courtesy of administration person	4.81	0.394	0	0.0	0	0.0	0	0.0	19	19.0	81	81.0
**Domain 2: Child’s room**												
Room appearance	4.78	0.579	1	1.0	0	0.0	2	2.0	14	14.0	83	83.0
Room cleanliness	4.79	0.518	1	1.0	0	0.0	2	2.0	14	14.0	83	83.0
How well things work	4.83	0.378	0	0.0	0	0.0	0	0.0	17	17.0	83	83.0
Courtesy of housekeeper	4.79	0.537	0	0.0	0	0.0	6	6.0	9	9.0	85	85.0
**Domain 3: Meals**												
Explanation of the child’s diet	4.46	0.658	0	0.0	0	0.0	9	9.0	36	36.0	55	55.0
Temperature of the food	4.29	0.556	0	0.0	1	1.0	2	2.0	64	64.0	33	33.0
Quality of the food	4.22	0.824	1	1.0	0	0.0	19	19.0	36	36.0	44	44.0
Availability of the type of food that the child likes	4.19	0.706	0	0.0	1	1.0	14	14.0	50	50.0	35	35.0
**Domain 4: Care from nurses**												
Friendliness/courtesy of the nurses	4.97	0.171	0	0.0	0	0.0	0	0.0	3	3,0	97	97.0
Promptness in responding to the call bell	4.87	0.418	0	0.0	0	0.0	3	3.0	7	7.0	90	90.0
Nurses’ attitude towards your requests	4.85	0.435	0	0.0	0	0.0	3	3.0	9	9.0	88	88.0
Amount of attention paid to your child’s special/personal needs	4.83	0.378	0	0.0	0	0.0	0	0.0	17	17.0	83	83.0
Degree to which nurses kept you informed using language you can understand	4.87	0.338	0	0.0	0	0.0	0	0.0	13	13.0	87	87.0
Skills of the nurses	4.83	0.473	0	0.0	0	0.0	4	4.0	9	9.0	87	87.0
Identity band check prior to giving medication	4.90	0.302	0	0.0	0	0.0	0	0.0	10	10.0	90	90.0
**Domain 5: Anaesthesia (*n* = 38)**												
Explanation provided by the anaesthesiologist	4.68	0.66	0	0.0	0	0.0	1	3.0	11	29.0	26	68.0
Friendliness/courtesy of the anaesthesiologist	4.79	0.59	0	0.0	0	0.0	1	3.0	6	16.0	31	81.0
Staff’s sensitivity and responsiveness to your child’s pain in the RR	4.71	0.52	0	0.0	0	0.0	0	0.0	11	29.0	27	71.0
Your rating of the anaesthesia service	4.61	0.77	0	0.0	0	0.0	1	3.0	13	34.0	24	63.0
**Domain 6: Tests and treatments**												
Skills of the person who took your child’s blood	4.67	0.533	0	0.0	0	0.0	3	3.0	27	27.0	70	70.0
Skills of the person who started the IV	4.75	0.500	0	0.0	0	0.0	3	3.0	19	19.0	78	78.0
Concern for your child’s comfort during tests, appointments and treatment	4.85	0.359	0	0.0	0	0.0	0	0.0	15	15.0	85	85.0
Degree to which tests, appointments and treatment were explained using language you could understand	4.64	0.578	0	0.0	1	1.0	2	2.0	29	29.0	68	68.0
**Domain 7: Family and visitors**												
Helpfulness of the people at the information desk	4.81	0.394	0	0.0	0	0.0	0	0.0	19	19.0	81	81.0
Staff’s attitude towards visitors	4.89	0.314	0	0.0	0	0.0	0	0.0	11	11.0	89	89.0
Comfort of the overnight facilities for parents	4.91	0.288	0	0.0	0	0.0	0	0.0	9	9.0	91	91.0
Information provided about available facilities for close family members	4.88	0.327	0	0.0	0	0.0	0	0.0	12	12.0	88	88.0
**Domain 8: Child’s physician**												
Time the physician spent with your child	4.69	0.526	0	0.0	0	0.0	3	3.0	25	25.0	72	72.0
Degree to which the physician kept you informed	4.85	0.359	0	0.0	0	0.0	0	0.0	15	15.0	85	85.0
Physician’s concern for your and your child’s questions and worries	4.93	0.256	0	0.0	0	0.0	0	0.0	7	7.0	93	93.0
How friendly and caring the physician was towards your child	4.95	0.219	0	0.0	0	0.0	0	0.0	5	5.0	95	95.0
Trust you had in your child’s physician	4.98	0.141	0	0.0	0	0.0	0	0.0	2	2.0	98	98.0
**Domain 9: Discharge**												
Degree to which you felt ready to have your child discharged.	4.72	0.451	0	0.0	0	0.0	0	0.0	28	28.0	72	72.0
Speed of the discharge process after you were told your child could go home	4.76	0.452	0	0.0	0	0.0	1	1.0	22	22.0	77	77.0
Instruction given about how you care for your child at home	4.80	0.426	0	0.0	0	0.0	1	1.0	18	18.0	81	81.0
**Domain 10: Personal issues**												
Staff’s concern for your and your child’s privacy	4.89	0.345	0	0.0	0	0.0	1	1.0	9	9.0	90	90.0
Degree to which the hospital staff addressed your emotional needs	4.34	0.755	0	0.0	1	1.0	14	14.0	35.0	25.0	50	50.0
Response to concerns/complaints made during your child’s stay	4.82	0.411	0	0.0	0	0.0	1	1,0	16	16.0	83	83.0
Staff’s efforts to include you in decisions about your child’s treatment	4.83	0.378	0	0.0	0	0.0	0	0.0	17	17.0	83	83.0
Degree to which staff respected your knowledge of your own child	4.93	0.256	0	0.0	0	0.0	0	0.0	7	7.0	93	93.0
Staff’s concern not to frighten your child	4.83	0.378	0	0.0	0	0.0	0	0.0	17	17.0	83	83.0
How well your child’s pain was controlled	4.80	0.402	0	0.0	0	0.0	0	0.0	20	20.0	80	80.0
Staff’s concern to make your child’s stay as restful as possible	4.89	0.314	0	0.0	0	0.0	0	0.0	11	11.0	89	89.0
Medical staff’s hand hygiene before examination	4.65	0.575	0	0.0	0	0.0	5	5.0	25	25.0	70	70.0
**Domain 11: Overall assessment**												
Overall cheerfulness of the hospital	4.62	0.508	0	0.0	0	0.0	1	1.0	36	36.0	63	63.0
How well staff worked together to care for your child	4.80	0.402	0	0.0	0	0.0	0	0.0	20	20.0	80	80.0
Overall rating of the care given at the hospital	4.95	0.219	0	0.0	0	0.0	0	0.0	5	5.0	95	95.0
Likelihood of you recommending this hospital to others	4.92	0.273	0	0.0	0	0.0	0	0.0	8	8.0	92	92.0

s.d., standard deviation; RR, recovery room; IV, Intravenous Access.

### Qualitative Insights: Caregiver experiences

To complement the survey results, nine in-depth interviews were conducted with caregivers, revealing six major themes:

1. *Care from Nurses*: Families appreciated professionalism, compassion and attentiveness.
2. *Care from Physicians*: Trust in physicians and clear communication were key positive aspects.
3. *Positive Experiences*: Participants praised hospital facilities and the support received.
4. *Negative Experiences*: Delays in services and a lack of specialised paediatric urologists were concerns.
5. *Areas for Improvement*: Recommendations included family education, enhanced emotional support, infrastructure development and better meal options.
6. *Emotional Response*: Caregivers described their experience as ‘difficult’ and ‘fearful’, but also expressed ‘gratitude’ and ‘acceptance’.

Table 5 summarises the key qualitative themes.

**TABLE 5:** Summary of the categories and subcategories that emerged from the qualitative data.

Categories	Subcategories
**Question one: ‘In your journey through the oncology units, can you describe your and your child’s experiences with care?’ Probe ‘What are the things that you liked and things you disliked?’**	
Care from the nurses	1.1 Professionalism, competence and skills
	1.2 Compassionate and caring staff
	1.3 Nurses provide excellent care
Care from the physicians	2.1 Trust in the physicians
	2.2 Communication from the physicians
	2.3 Consistent visits
Positive experience	3.1 Excellent care and service
	3.2 Satisfaction with hospital facilities
	3.3 Emotional or psychological support from the medical team
Negative experience	4.1 Negative experience with EMS
	4.4 Service delays
	4.5 Unavailability of paediatric urologists
**Question two: ‘Can you suggest ways that we could improve your care?’**	
Improvement in care	5.1 Family education
	5.2 Infrastructural improvement
	5.3 Humane care and emotional support
	5.4 Social activities
	5.5 Meal improvement
	5.6 Policy alignment
**Question three: ‘I would like you to tell me a word or a phrase that summarises your feelings through your journey. I would ask you to tell me the first one that comes to mind’.**	
Feelings during the journey stages	6.1 Difficult, painful, sad and traumatic
	6.2 Fear and uncertainty
	6.3 Religious acceptance and gratitude
	6.4 Feeling overwhelmed
**Question four: ‘Are you given an option based on the type of food that your child likes?’**	
Meal quality	7.1 Dissatisfaction with meals
	7.2 Satisfaction with meals

EMS, Emergency Medicine Service.

By merging the quantitative satisfaction scores with qualitative narratives, key areas for intervention emerged:

- *Communication at Diagnosis Stage*: Families reported high distress because of unclear explanations. The journey map incorporated structured communication checkpoints.
- *Emotional Support Needs*: Post-discharge challenges, including limited access to palliative care, were flagged. The journey map integrated scheduled follow-ups and psychological support.
- *Service Gaps*: Families experienced difficulties in meal preferences and discharge education, reinforcing the need for service optimisation.

#### Stage 2: Refinement using mixed-method integration

The initial journey map was refined by overlaying quantitative survey results and qualitative insights to ensure real-world applicability as presented in Table 6.

**TABLE 6:** Patients’ and families’ experiences at each stage of the patient’s journey from the integration of the quantitative and qualitative findings.

Category	Pre-diagnosis	Diagnosis	Treatment	Discharge	Post-discharge follow-up
Positives	Effective communication by physician	Effective admission personnel Trust in the medical team Availability of oncology specialised facility within the region ‘I appreciate that they called me and my wife for a meeting when my son was first diagnosed with cancer’. ‘The good thing however is that we don’t have to travel to Riyadh for his chemo. Like today, we can have his treatment here, so I am happy about that.’	Consistent visit by physicians Emotional and psychological support provided Compassionate care provided Satisfactory care from nurses and MDs ‘Doctor visits every day’ ‘Staff are very caring and compassionate towards us and always willing to help’	Satisfaction with hospital facilities Excellent care and service	-
Negatives	Fear and anxiety ‘Is everything going to be okay?’	Service delays Lack of paediatric specialised nurse in ED	Lack of variety in the meals Unavailability of paediatric urologist High sink in the bathrooms		Insufficient quantity and poor quality of food served in outpatient oncology area
Feeling	Fear and anxiety ‘Is everything going to be okay?’	Difficult Painful Sad ‘I had a hard time accepting the facts about the disease.’ ‘I can’t bear to see my child suffer.’	Overwhelmed Traumatic Uncertain Anxiety in OR ‘Will the chemo cure my son’s cancer?’	Religious acceptance and gratitude ‘Praise be to God in all situations’ ‘With hardship, there will be ease’	Appreciation Gratitude ‘I am satisfied with the services throughout our hospital journey.’ ‘We receive excellent service, and I would like to thank all medical team’
Improvement in care	Arrangement of work-up; lack of slots; lack of manpower; lack of specified equipment or facility	Human care and emotional support Preference for paediatric specialised nurse in ER ‘I think everyone needs to understand what it is to be human. To be able to empathize with patients.’ ‘But we prefer to be in the ward because they have more expert nurses who are specialized with small kids.’	Uniformity of communication and policy alignment Education and reassurance at treatment commencement Meal improvement ‘At the beginning of the treatment, it would be helpful if the family can be given extra education and a reassurance that there is hope so the family doesn’t lose hope or despair.’	More social activities for the kids Improvement of meals quality Facility or infrastructural improvementLonger visiting hours ‘The social department plays a major role in keeping the environment happy and positive for the kids and they should be involved in ensuring that the patients are happy in the hospital environment.’	Improvement of meals in the outpatient oncology area

ED, Emergency Department; ER, Emergency Room; MD, physician; OR, Operating Room.

#### Stage 3: Validation through the modified Delphi approach

**FIGURE 1:**
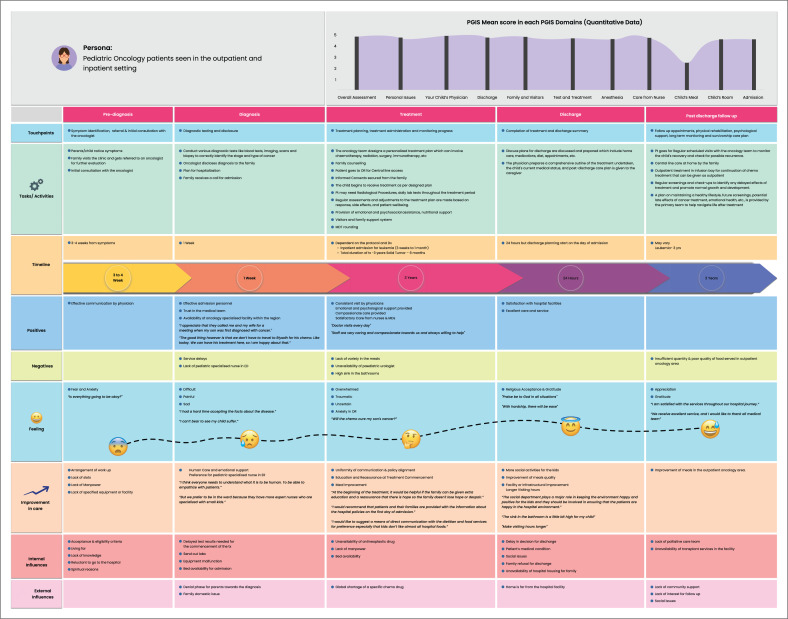
Paediatric oncology journey map design (https://drive.google.com/file/d/14WAt-j-BQlUK6aXAdfYuhiFu5CcTd2qJ/view?usp=sharing)

The final phase of the journey mapping process involved refining the patient journey map prototype. The researcher adapted the modified Delphi technique for this study from the Delphi method as utilised by Ly, Runacres and Poon (2021), which incorporated two iterations of questionnaire distribution. Email invitations were dispatched to the senior oncology staff, including four senior oncology nurses and two paediatric oncology physicians who had earlier participated in the workshop phase of the research and had volunteered to be part of the Delphi. These emails encompassed an explanatory statement, the journey map design file link https://drive.google.com/file/d/14WAt-j-BQlUK6aXAdfYuhiFu5CcTd2qJ/view?usp=sharing (Figure 1) and a link to the questionnaire.

### Final patient journey map: Key features

The finalised paediatric oncology patient journey map is a visual representation of a patient’s care experience, designed to improve clinical workflows by aligning care processes with patient needs, enhance caregiver support by providing clear guidance on what to expect at each stage and identify service gaps for targeted quality improvement initiatives.

This structured, finalised patient journey map serves as a tool for optimising paediatric oncology care, ensuring that healthcare providers, patients and caregivers are better informed and supported throughout the care continuum.

## Discussion

This article aimed to describe the methodological process of developing a paediatric oncology patient journey map and to present integrated insights from families’ experiences across different stages of care. The discussion therefore addresses not only how the map was constructed but also the lessons it provides for improving patient–family experiences and care delivery in practice. Family members highlighted key challenges related to communication, emotional burden and logistical difficulties during different stages of the oncology journey. One of the most pressing concerns was the lack of clear communication at the pre-diagnosis and diagnosis stages, where families expressed distress because of delayed or unclear explanations from medical teams. This finding aligns with existing literature emphasising the critical role of early-stage communication in paediatric oncology (Newman, Linder & Haglund 2020).

In response, the journey map incorporated structured communication touchpoints, ensuring caregivers receive timely and transparent information regarding their child’s diagnosis and treatment options. Additionally, emotional support was identified as a key gap, particularly during the transition from inpatient to post-discharge care. Many families reported feeling abandoned after discharge, with limited access to palliative care or community-based follow-up services. To address this, the journey map includes a post-discharge follow-up protocol, including scheduled check-ins and referral pathways to psychological support services.

### The role of the journey map in enhancing care pathways

Patient journey mapping has been widely recognised as a tool for identifying service inefficiencies and improving patient-centred care (Arias et al., 2020). By systematically analysing both patient experiences, this study’s journey map serves as a strategic framework for optimising paediatric oncology services. The integration of evidence-based strategies within the map aligns with best practices in healthcare service improvement, as highlighted in studies by Halvorsrud, Kvale and Følstad (2016) and Sijm-Eeken, Zheng and Peute (2020).

A key strength of the developed journey map is its adaptive static models, as this map was iteratively refined through stakeholder feedback, ensuring alignment with real-world clinical workflows and patient needs. The involvement of healthcare providers in the mapping process facilitated the identification of logistical bottlenecks, such as drug shortages and administrative delays, which were subsequently addressed in the final version.

The findings of this study align with previous research emphasising the utility of PJM in oncology care. A study by Agarwal et al. (2023) mapped the care pathways of lung and breast cancer patients to identify unmet needs and gaps in service delivery. Similarly, Kushniruk, Borycki and Parush (2020) highlighted how journey mapping enhances service improvement efforts by visually depicting inefficiencies in care coordination.

Moreover, Philpot et al. (2019) demonstrated that institutions implementing frameworks observed significant improvements in patient satisfaction and clinical outcomes. This study’s findings support this assertion, as the journey map provided a structured approach to mitigating distress and enhancing patient experience at each stage of care.

## Limitations

This article has several limitations that must be acknowledged. Firstly, as it focuses on the development and reporting of a patient journey map in a single tertiary hospital in the Kingdom of Saudi Arabia (KSA), the findings may not be generalisable to other healthcare systems with different resources, policies or patient populations. Secondly, the journey map primarily reflects the perspectives of caregivers and family members, which, while valuable, may not fully represent the direct experiences of paediatric oncology patients themselves. Thirdly, the insights presented are descriptive and illustrative of the care journey, but the article does not evaluate the practical implementation or effectiveness of using the journey map in clinical workflows. Fourthly, although the integration of quantitative and qualitative data strengthened the map’s development, qualitative findings are inherently subjective and may be influenced by recall or response bias. Fifthly, given the researcher’s affiliation with the study hospital, the potential for bias cannot be completely excluded, although bracketing and triangulation were applied to enhance objectivity. While the patient journey map developed in this study provides a structured understanding of the care pathway, it may not capture all aspects of the patient experience, particularly emotional and psychological distress, which may require further exploration.

### Recommendations

The findings suggest several directions for practice and research. Incorporating structured communication at critical stages of care and strengthening psychosocial and post-discharge support would address gaps identified by families. The journey map developed here can serve as a framework for staff training, quality improvement initiatives and improved coordination of care. Journals and researchers are encouraged to further test and adapt this tool in different paediatric oncology settings, broadening its utility and transferability.

## Conclusion

This study provides a comprehensive analysis of the paediatric oncology patient journey using a mixed-methods approach, integrating both qualitative and quantitative findings. The development of the patient journey map offers a structured framework for understanding key touchpoints in the care pathway, identifying strengths and highlighting areas for improvement. The findings indicate that while families generally reported positive experiences regarding medical care and staff interactions, challenges related to emotional support, communication and logistical aspects such as hospital meals and post-discharge care remain. The study contributes to the growing body of literature supporting the use of PJM as a tool for healthcare quality improvement. It underscores the need for a patient-centred approach that incorporates both the clinical and emotional needs of paediatric oncology patients and their families. Future research should explore ways to refine the journey map by incorporating direct patient perspectives and expanding the study across multiple institutions to enhance generalisability.
